# [μ_2_-*trans*-1,2-Bis­(pyridin-4-yl)ethene-κ^2^ *N*:*N*′]bis­{[1,2-bis­(pyridin-4-yl)ethene-κ*N*]bis­[*N*-(2-hydroxy­eth­yl)-*N*-iso­propyl­dithio­carbamato-κ^2^ *S*,*S*′]cadmium} aceto­nitrile tetra­solvate: crystal structure and Hirshfeld surface analysis

**DOI:** 10.1107/S2056989016010768

**Published:** 2016-07-12

**Authors:** Mukesh M. Jotani, Pavel Poplaukhin, Hadi D. Arman, Edward R. T. Tiekink

**Affiliations:** a Department of Physics, Bhavan’s Sheth R. A. College of Science, Ahmedabad, Gujarat 380 001, India; b Chemical Abstracts Service, 2540 Olentangy River Rd, Columbus, Ohio 43202, USA; c Department of Chemistry, The University of Texas at San Antonio, One UTSA Circle, San Antonio, Texas 78249-0698, USA; d Centre for Crystalline Materials, Faculty of Science and Technology, Sunway University, 47500 Bandar Sunway, Selangor Darul Ehsan, Malaysia

**Keywords:** crystal structure, di­thio­carbamate, 1,2-bis­(pyridin-4-yl)ethene, inter­penetration, hydrogen bonding, Hirshfeld surface analysis

## Abstract

The asymmetric unit of the title compound, [Cd_2_(C_12_H_10_N_2_)_3_(C_6_H_12_NOS_2_)_4_]·4C_2_H_3_N, comprises a Cd^II^ atom, two di­thio­carbamate (dtc) anions, one and a half *trans*-1,2-dipyridin-4-yl­ethyl­ene (bpe) mol­ecules and two aceto­nitrile solvent mol­ecules. The full binuclear complex is generated by the application of a centre of inversion. The dtc ligands are chelating, one bpe mol­ecule coordinates in a monodentate mode while the other is bidentate bridging. The resulting *cis*-N_2_S_4_ coordination geometry is based on an octa­hedron. Supra­molecular layers, sustained by hy­droxy-O—H⋯O(hy­droxy) and hy­droxy-O—H⋯N(bpe) hydrogen bonding, inter­penetrate to form a three-dimensional architecture; voids in this arrangement are occupied by the aceto­nitrile solvent mol­ecules. Additional inter­molecular inter­actions falling within the specified framework have been analysed by Hirshfeld surface analysis, including π–π inter­actions.

## Abstract 2

A distorted octa­hedral *cis*-N_2_S_4_ coordination geometry is found in the title solvated bimetallic complex. The packing features supra­molecular layers sustained by O—H⋯O, O—H⋯N and N—H⋯O hydrogen bonding.

## Chemical context   

The recent disclosure of one-dimensional, supra­molecular isomers of {Cd[S_2_CN(*i*Pr)CH_2_CH_2_OH]_2_}_*n*_ notwithstanding (Tan *et al.*, 2013 ▸, 2016 ▸), the overwhelming majority of binary bis­(di­alkyl­dithio­carbamato) compounds of cadmium are usually binuclear with a coordination number of five owing to the presence of equal numbers of chelating and μ_2_-tridentate ligands, *i.e*. are of general formula [Cd(S_2_CN*R*_2_)_2_]_2_ (Tiekink, 2003 ▸; Tan *et al.*, 2016 ▸). However, the dimeric and polymeric aggregates are readily broken down in the presence of bases such as monodentate pyridine, *e.g*. {Cd[S_2_CN(CH_2_C(H)Me_2_)_2_]_2_(pyridine)} (Rodina *et al.*, 2011 ▸) and bidentate 2,2′-bi­pyridine, *e.g*. [Cd(S_2_CN(Me)*i*Pr)_2_(2,2′-bi­pyridine)] (Wahab *et al.*, 2011 ▸). Bridging N-donors lead to a greater variety of structures such as the zero-dimensional binuclear compound, [Cd(S_2_CNPr_2_)_2_(2-pyridine­aldazine)]_2_ (Poplaukhin & Tiekink, 2008 ▸) and supra­molecular chains, *e.g*. [Cd(S_2_CNEt_2_)_2_(μ_2_-1,2-bis­(4-pyrid­yl)ethyl­ene)]_*n*_ (Chai *et al.*, 2003 ▸). The addition of hydrogen-bonding functionality in the di­thio­carbamate ligands has greatly enhanced the supra­molecular chemistry landscape of related compounds. As a recent exemplar, the formally monomeric compound {Cd[S_2_CN(*i*Pr)CH_2_CH_2_OH]_2_}(piperazine) self-assembles into a two-dimensional array *via* hy­droxy-O—H⋯O(hy­droxy), hy­droxy-O—H⋯N(terminal-piperazine) and coordinating piperazine-N—H⋯O(hy­droxy) hydrogen bonds (Safbri *et al.*, 2016 ▸). As a continuation of investigations in this area, the crystal and mol­ecular structure as well as Hirshfeld surface analysis of the title binuclear compound, {Cd[S_2_CN(*i*Pr)CH_2_CH_2_OH]_2_[(4-NC_5_H_4_)C=C_6_H_4_N-4)]}_2_[(4-NC_5_H_4_)C=C_6_H_4_N-4)]·4CH_3_CN, (I), featuring both bidentate bridging and monodentate *trans*-1,2-dipyridin-4-yl­ethyl­ene ligands is described herein.
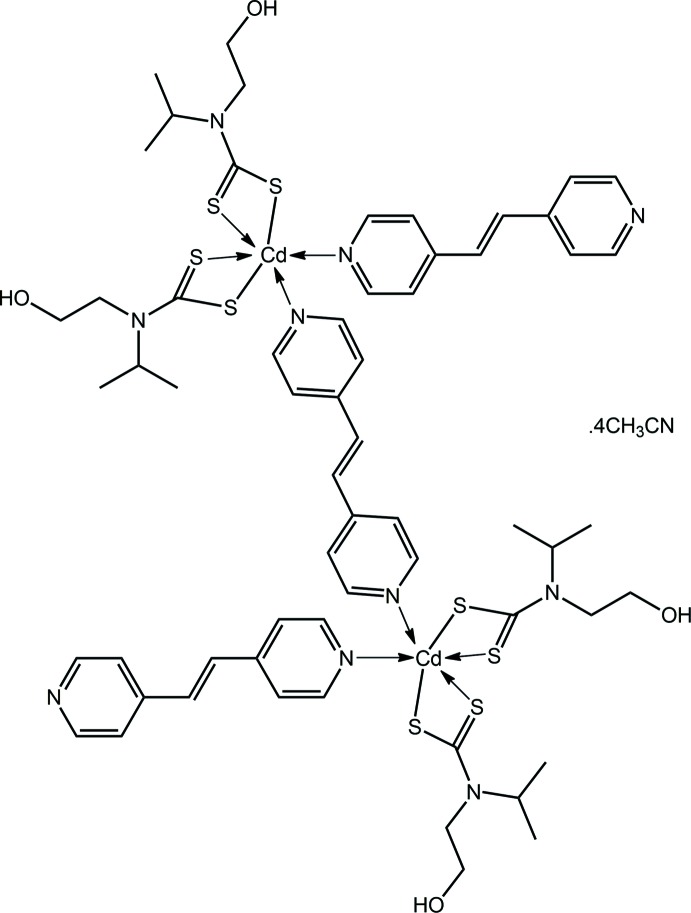


## Structural commentary   

**Figure 1:**
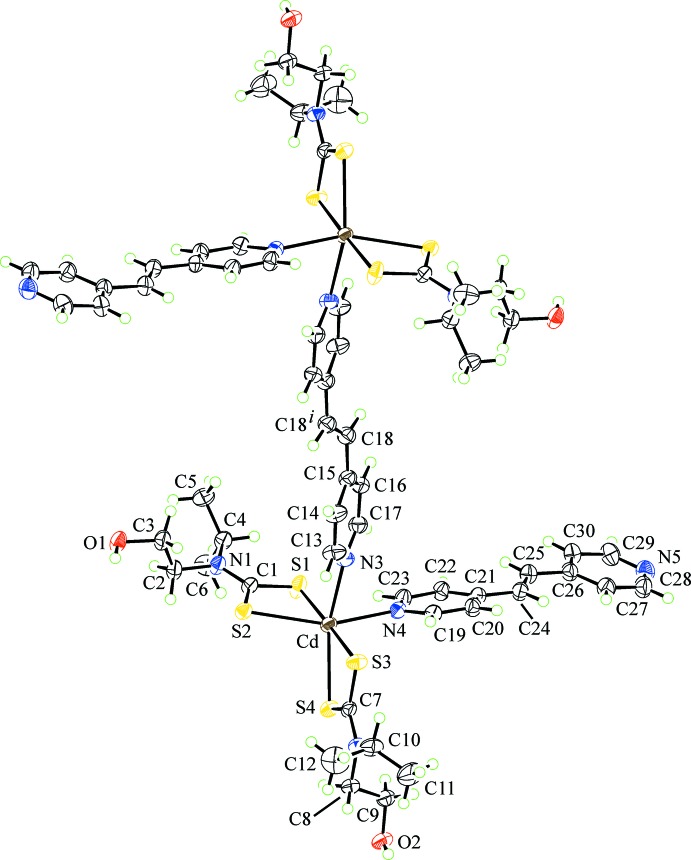
The mol­ecular structure of the binuclear title compound in (I), showing the atom-labelling scheme and displacement ellipsoids at the 50% probability level. Unlabelled atoms are related by the symmetry operation (2 − *x*, −*y*, 1 − *z.*). The aceto­nitrile solvent mol­ecules have been omitted for clarity.

The mol­ecular structure of the binuclear title compound, {Cd[S_2_CN(*i*Pr)CH_2_CH_2_OH]_2_[(4-NC_5_H_4_)C=C_6_H_4_N-4)]}_2_[(4-NC_5_H_4_)C=C_6_H_4_N-4)]·4CH_3_CN, (I), Fig. 1 ▸, is situated about a centre of inversion; two aceto­nitrile mol­ecules of solvation complete the asymmetric unit. Each Cd^II^ atom is coordinated by two di­thio­carbamate ligands and two nitro­gen atoms, one derived from a monodentate *trans*-1,2-dipyridin-4-yl­ethyl­ene (bis­pyridyl­ethene; bpe) ligand and another from one end of a bidentate, bridging bpe ligand (located about a centre of inversion). The di­thio­carbamate ligands coordinate with significant differences in their Cd—S bond lengths, Table 1 ▸. Thus, Δ(Cd—S) = *d*(Cd—S_long_) – *d*(Cd—S_short_) = 0.15 Å for the S1-di­thio­carbamate ligand *cf*. 0.10 Å for the S3-ligand. Nevertheless, there is considerable delocalization of π-electron density in the CdS_2_C chelate rings as evidenced by the equivalence of the associated C—S bond lengths, Table 1 ▸. The coordination geometry is based on an octa­hedron. In this description, the more tightly bound S1 and S3 atoms are *trans* [178.06 (3)°] and the less tightly bound sulfur atoms are *trans* to nitro­gen atoms, Table 1 ▸, implying the nitro­gen donors are *cis*. The distortions from the ideal geometry are readily related to the restricted bite angles of the chelating ligands, Table 1 ▸. Both bpe ligands exhibit twists as seen in the values of the C14—C15—C18—C18^i^ and C22—C21—C24—C25 torsion angles of −12.2 (6) and 13.9 (5)° for the bi- and mono-dentate ligands, respectively; symmetry code: (i) 2 − *x*, −*y*, 1 − *z*.

## Supra­molecular features   

**Figure 2:**
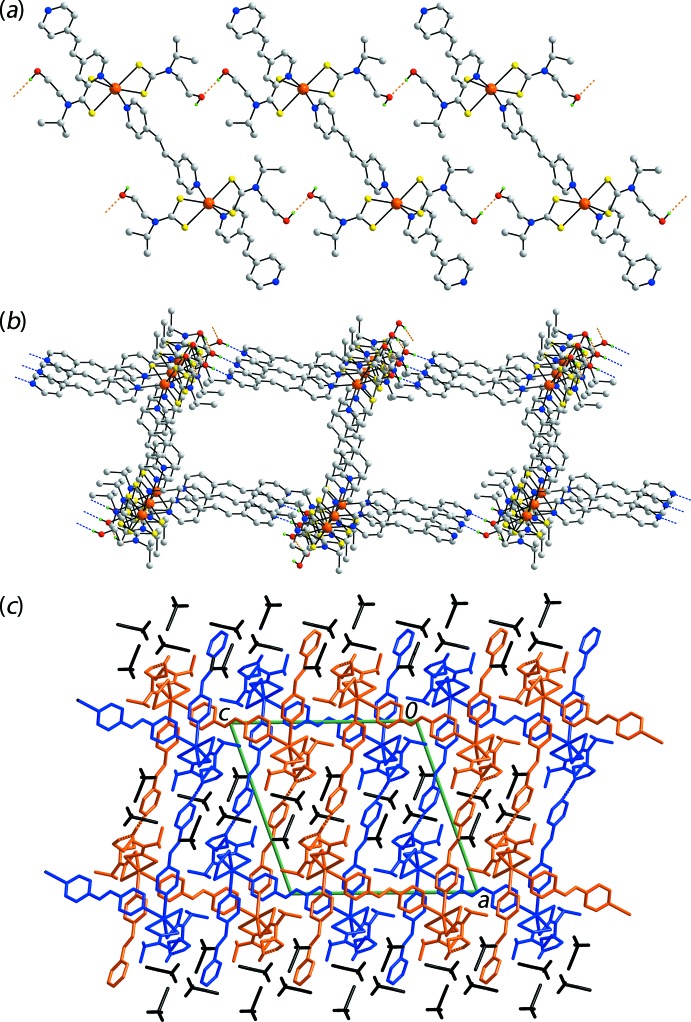
Mol­ecular packing in (I): (*a*) view of the supra­molecular ladder sustained by hy­droxy-O—H⋯O(hy­droxy) hydrogen bonds, shown as orange dashed lines, (*b*) two-dimensional framework whereby the layers in (*a*) are connected by hy­droxy-O—H⋯N(bpe) hydrogen bonds, shown as blue dashed lines, (*c*) view of the unit-cell contents shown in projection down the *a* axis, highlighting the inter­penetration of successive supra­molecular layers, illustrated in orange and green, with solvent aceto­nitrile mol­ecules shown in black.

Geometric details of the significant inter­molecular inter­actions are given in Table 2 ▸. In the packing, hy­droxy-O—H⋯O(hy­droxy) hydrogen bonding leads to supra­molecular ladders as illustrated in Fig. 2 ▸ *a*. These ladders are connected into layers parallel to (101) *via* hy­droxy-O—H⋯N(bpe) hydrogen bonds where the nitro­gen atom is derived from the monodentate bpe ligand. Additional ethene-C—H⋯O(hy­droxy) inter­actions are found within this framework, Table 2 ▸. As seen from Fig. 2 ▸ *b*, this arrangement leads to rectangular channels with Cd⋯Cd separations, which approximate the edges, being 14 and 16 Å. Successive channels are largely occupied by other supra­molecular layers, leading to a three-dimensional, concatenated architecture. The smaller voids defined by the inter­penetrated structure are occupied by the solvent aceto­nitrile mol­ecules, Fig. 2 ▸ *c*. The N7-aceto­nitrile mol­ecule is connected to the host framework by pyridyl-C—H⋯N(aceto­nitrile) inter­actions whereas the N6-aceto­nitrile mol­ecule does not form significant inter­actions in accord with the criteria embodied in *PLATON* (Spek, 2009 ▸). This is reflected in the greater displacement ellipsoids for this mol­ecule *cf*. with the N7-containing mol­ecule. Further analysis of the mol­ecular packing, *e.g*. pyrid­yl⋯pyridyl inter­actions, is given in the following Section.

## Analysis of the Hirshfeld surfaces   

Recent Hirshfeld surface analyses of zinc-triad hy­droxy­ethyl-substituted di­thio­carbamates has provided key insight into their mol­ecular packing over and beyond hydrogen-bonding considerations. For example, the relatively unusual C—H⋯π(chelate) inter­actions (Tiekink & Zukerman-Schpector, 2011 ▸) observed in [Hg(S_2_CN(CH_2_CH_2_OH)_2_]_*n*_ (Howie *et al.*, 2009 ▸), are clearly delineated in the Hirshfeld analysis of the mol­ecular packing (Jotani *et al.*, 2016 ▸). In the present study, using *Crystal Explorer 3.1* (Wolff *et al.*, 2012 ▸), the Hirshfeld surfaces were mapped over *d*_norm_, shape-index, curvedness and electrostatic potential for the asymmetric unit of (I). The electrostatic potentials were calculated using *TONTO* (Spackman *et al.*, 2008 ▸; Jayatilaka *et al.*, 2005 ▸) integrated into *Crystal Explorer*. Further, the electrostatic potentials were mapped on Hirshfeld surfaces using the STO–3G basis set at Hartree–Fock level of theory over a range ±0.13 au. The contact distances *d*_i_ and *d*_e_ from the Hirshfeld surface to the nearest atom inside and outside, respectively, enable the analysis of the inter­molecular inter­actions through the mapping of *d*_norm_. The combination of *d*_e_ and *d*_i_ in the form of two-dimensional fingerprint plots (McKinnon *et al.*, 2004 ▸) provides a summary of inter­molecular contacts in the crystal.

**Figure 3:**
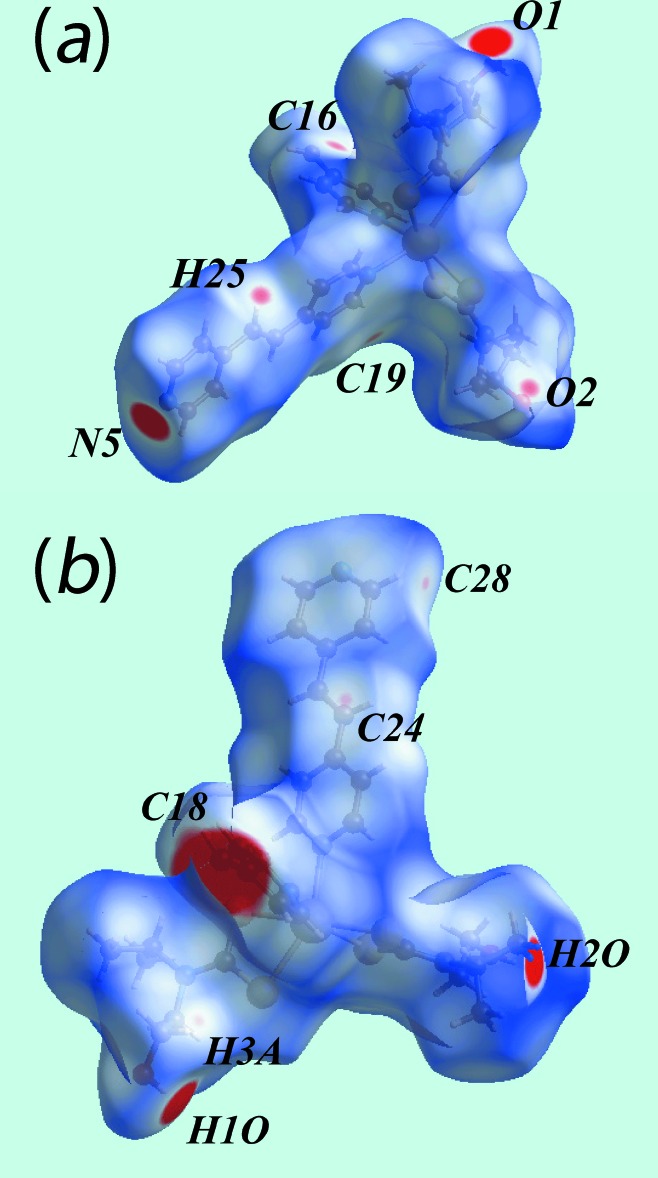
Two views of the Hirshfeld surface mapped over *d*_norm_. The contact points (red) are labelled to indicate the atoms participating in the inter­molecular inter­actions.

**Figure 4:**
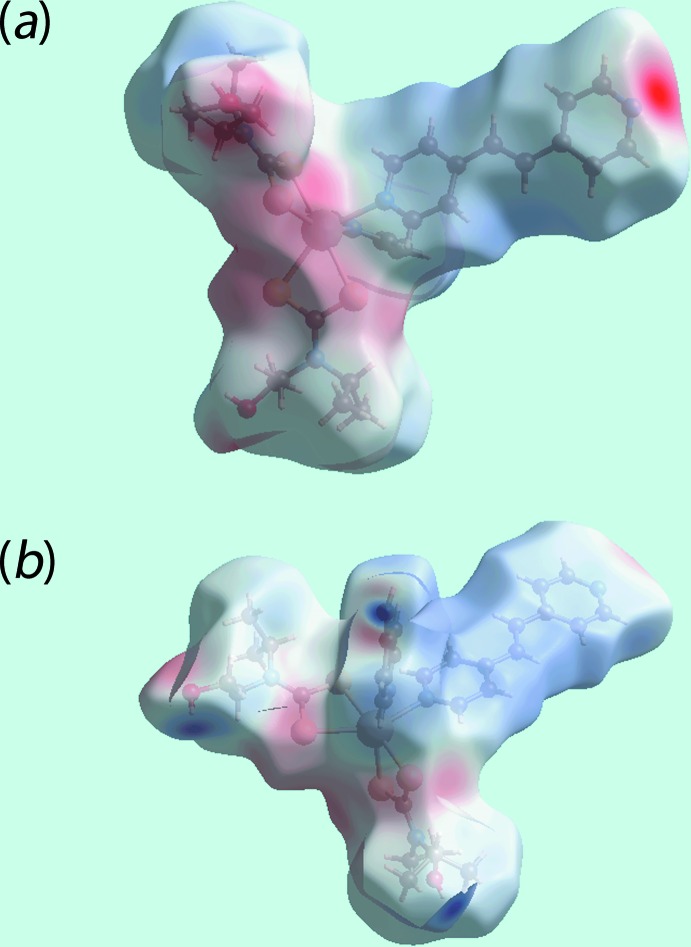
Two views of the Hirshfeld surface mapped over the electrostatic potential with positive and negative potential indicated in blue and red, respectively.

**Figure 5:**
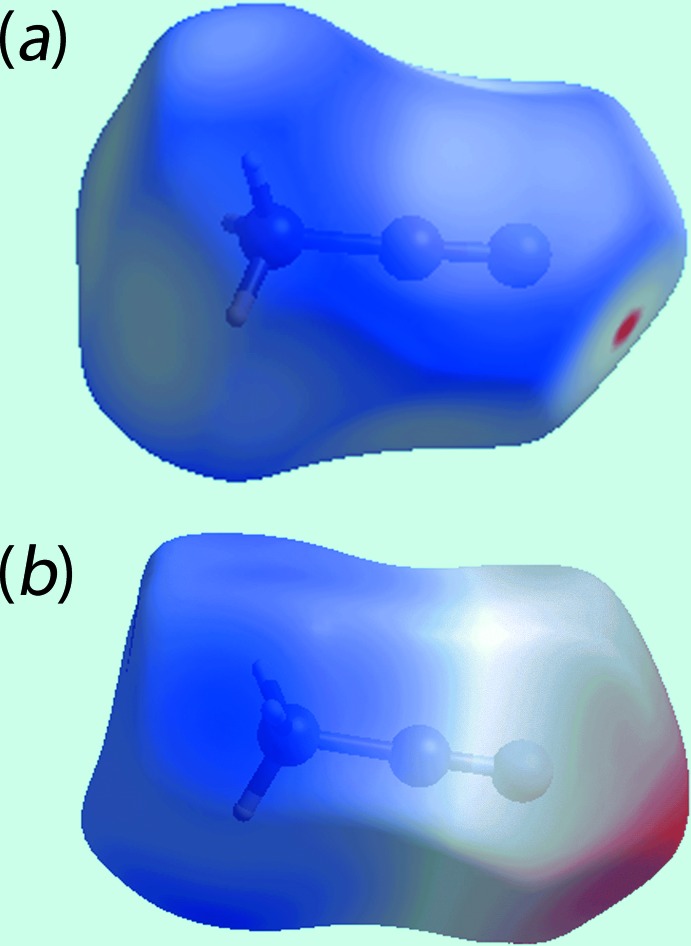
A view of the (*a*) Hirshfeld surface mapped over *d*_norm_ and (*b*) Hirshfeld surface mapped over the electrostatic potential with positive and negative potential indicated in blue and red, respectively, for the N7-aceto­nitrile mol­ecule.

Two views of Hirshfeld surfaces mapped over *d*_norm_ in the −0.2 to 1.8 Å range are shown in Fig. 3 ▸. The bright-red spots appearing near pyridyl-N5, hy­droxy-O1 and hydrogen atoms H1*O* and H2*O* indicate their role as the respective donors and acceptors in the dominant O—H⋯O and O—H⋯N hydrogen bonds; they also appear as blue and red regions, respectively, corresponding to positive and negative electrostatic potentials on the Hirshfeld surface mapped over electrostatic potential, Fig. 4 ▸. The light-red spots near ethene-H25, pyridyl-C28 and hy­droxy-O2 in Fig. 3 ▸ and near aceto­nitrile-N7, Fig. 5 ▸ *a*, indicate their involvement in the inter­molecular ethene-C—H⋯O(hy­droxy) and pyridyl-C—H⋯N(acetonitrile) inter­actions. The presence of short inter­molecular C⋯C and C⋯H contacts, Table 3 ▸, is also evident from the light-red spots appearing near the pyridyl-C16, C19 and C24 and methyl­ene-H3*A* atoms in Fig. 3 ▸. The C18—C18^i^ link of the bridging bpe ligand can be viewed as a bright-red region around the C18 atom in the *d*_norm_ mapped surface, Fig. 3 ▸, and as a light-blue region surrounded by a pair of light-red arcs on the surface mapped over electrostatic potential, Fig. 4 ▸ *b*; this arises as it is the asymmetric unit that has been investigated not the entire binuclear mol­ecule. With respect to the aceto­nitrile molecule the *d*_norm_ mapped surfaces show only the aceto­nitrile-N7 to be involved in a significant inter­molecular C—H⋯N inter­action (Fig. 5 ▸ *a*, Table 2 ▸), and both aceto­nitrile mol­ecules had very similar Hirshfeld surfaces mapped over electrostatic potential to that for the N7-mol­ecule illustrated in Fig. 5 ▸ *b*.

**Figure 6:**
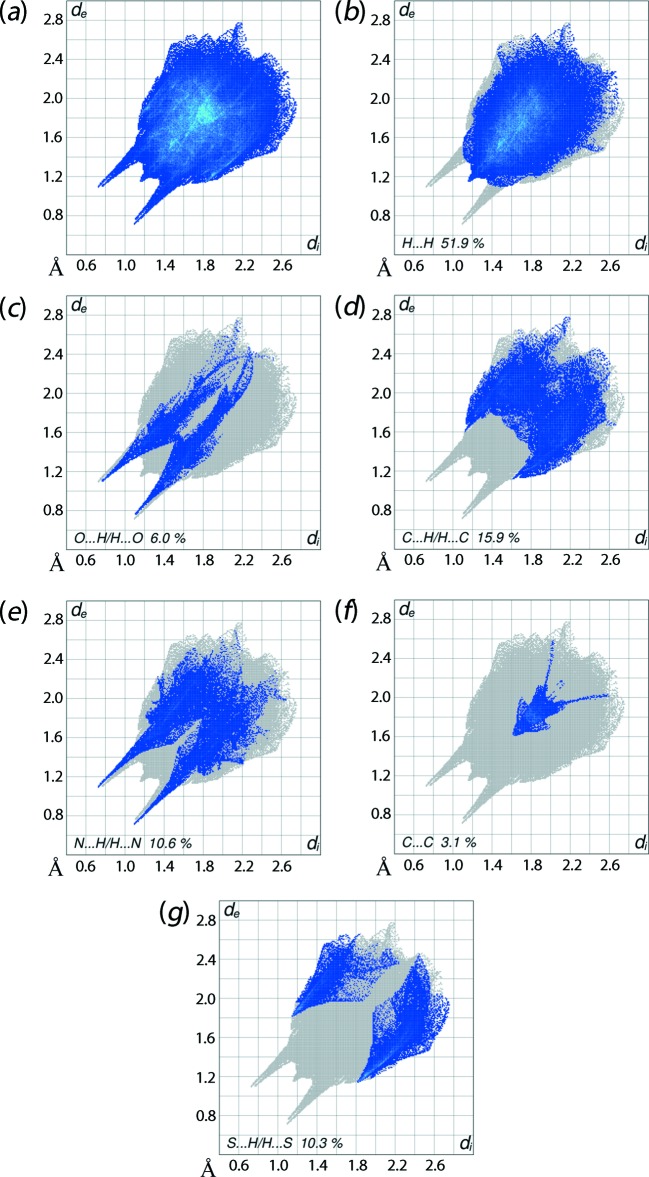
Two-dimensional fingerprint plots: (*a*) overall, and delineated into contributions from different contacts: (*b*) H⋯H, (*c*) O⋯H/H⋯O, (*d*) C⋯H/H⋯C, (*e*) N⋯H/H⋯N, (*f*) C⋯C and (*g*) S⋯H/H⋯S.

The overall two-dimensional fingerprint plot, Fig. 6 ▸ *a*, and those delineated into H⋯H, O⋯H/H⋯O, C⋯H/H⋯C, N⋯H/H⋯N, C⋯C and S⋯H/H⋯S contacts (McKinnon *et al.*, 2007 ▸) are illustrated in Fig. 6 ▸ *b*-*g*, respectively; their relative contributions are summarized in Table 4 ▸. The H⋯H contacts make the greatest contribution to the Hirshfeld surface, *i.e*. 51.9% which is reflected in Fig. 6 ▸ *b* as widely scattered points of high density due to the large hydrogen content of the mol­ecule; the single peak at *d*_e_ = *d*_i_ ∼1.15 Å results from a short inter­molecular H⋯H contact between the isopropyl-H5*A* and pyridyl-H23 atoms, Table 3 ▸. In the fingerprint plot delineated into O⋯H/H⋯O contacts, the 6.0% contribution to the Hirshfeld surface arises from the inter­molecular O—H⋯O hydrogen bonding and is viewed as a pair of spikes with the tip at *d*_e_ + *d*_i_ ∼1.8 Å in Fig. 6 ▸ *c*. The inter­molecular C—H⋯O inter­actions and short O⋯H/H⋯O contacts, listed in Table 3 ▸, are masked by the strong O—H⋯O hydrogen bonding in this plot.

**Figure 7:**
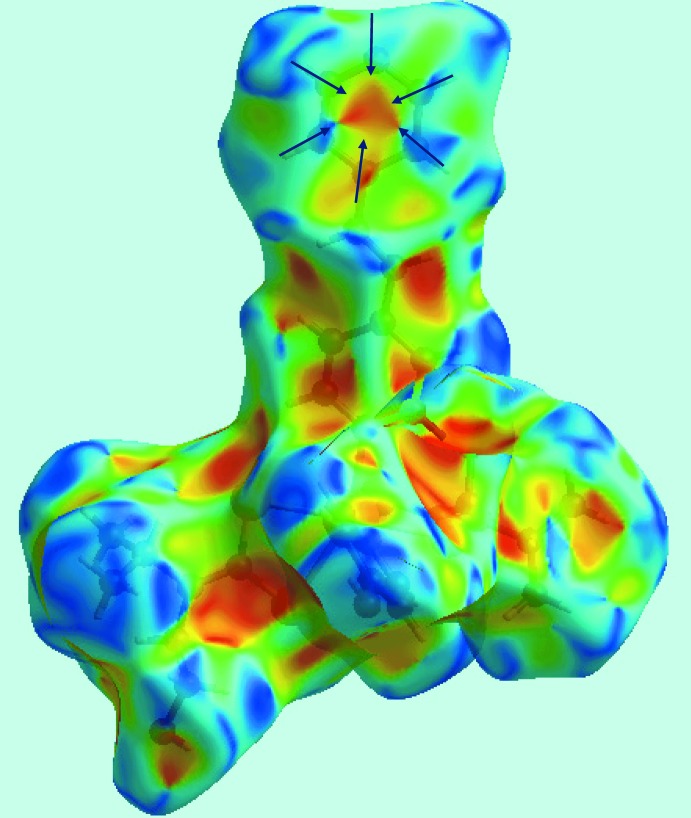
View of Hirshfeld surface mapped with shape-index property. The pairs of red and blue regions, identified with arrows, indicate π–π stacking inter­actions.

**Figure 8:**
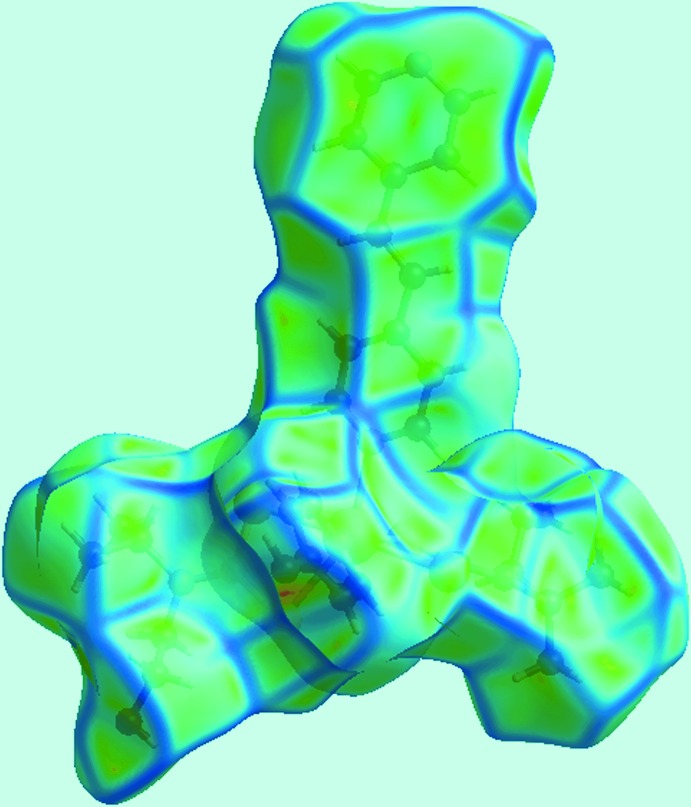
A view of Hirshfeld surface mapped over curvedness for (I). The flat regions highlight the involvement of rings in π–π stacking inter­actions.

In the absence of C—H⋯π inter­actions in the crystal, the pair of characteristic wings resulting in the fingerprint plot delineated into C⋯H/H⋯C contacts with 15.9% contribution to the Hirshfeld surface, Fig. 6 ▸ *d*, and the pair of thin edges at *d*_e_ + *d*_i_ ∼2.7 Å result from short inter­atomic C⋯H/H⋯C contacts, Table 3 ▸. A pair of spikes at *d*_e_ + *d*_i_ ∼1.8 Å correspond to N⋯H/H⋯N contacts, Fig. 6 ▸ *e*, confirm the presence of inter­molecular O—H⋯N and C—H⋯N inter­actions. The C⋯C contacts assigned to short inter­atomic C16⋯C19 and C16⋯C20 contacts listed in Table 3 ▸ and π–π stacking inter­actions within the three-dimensional architecture described in *Supra­molecular features* appear as the two distinct distributions of points in Fig. 6 ▸ *f*. The vertex at *d*_e_ = *d*_i_ = 1.6 Å in the approximately triangular distribution of points in the plot corresponds to short inter­molecular C⋯C contacts. The presence of π–π stacking inter­actions between the centrosymmetrically related N5-pyridyl rings [inter-centroid distance = 3.674 (2) Å, symmetry code: 3 − *x*, 1 − *y*, 2 − *z*] is reflected through the appearance of green points around *d*_e_ = *d*_i_ ∼1.8 Å, the red and blue triangle pairs on the Hirshfeld surface mapped with shape-index property identified with arrows in the image of Fig. 7 ▸, and in the flat region on the Hirshfeld surface mapped over curvedness in Fig. 8 ▸. Finally, the S⋯H/H⋯S contacts in the structure with a 10.3% contribution to the surface has a nearly symmetrical distribution of points, Fig. 6 ▸ *g*, with the tips at *d*_e_ + *d*_i_ ∼2.95 Å arising from the short inter­atomic S⋯H/H⋯S contacts listed in Table 3 ▸.

An additional descriptor, the enrichment ratio (ER), may be calculated on the basis of Hirshfeld surface analysis (Jelsch *et al.*, 2014 ▸). This provides further insight into the mol­ecular packing as it indicates the relative propensities to form specific inter­molecular inter­actions. The ER values for (I) are collected in Table 5 ▸. The ER value close to but slightly less than unity for H⋯H contacts, *i.e*. 0.97, is in accord with expectation (Jelsch *et al.*, 2014 ▸). The ER value of 1.36 for O⋯H/H⋯O contacts is in the expected 1.2–1.6 range and confirms the involvement of these atoms in the inter­molecular O—H⋯O and C—H⋯O inter­actions. The ER value of 1.20 resulting from the 6% of the surface comprising nitro­gen atoms and the 10.6% contribution to the Hirshfeld surface from N⋯H/H⋯N contacts is due to the presence of O—H⋯N hydrogen bonding and the C—H⋯N(aceto­nitrile) inter­action. The high enrichment ratio of 2.23 for the C⋯C contacts reflects the formation of significant π–π stacking inter­actions and short C⋯C contacts as mentioned above. The ER value close to unity, *i.e*. 0.92, for C⋯H/H⋯C contacts shows their propensity to form short inter­molecular C⋯H/H⋯C contacts. The ER values < 1 related to other contacts and low percentage contribution to the surface do not show any significance in the crystal packing.

## Database survey   

There is a sole example of a cadmium di­thio­carbamate coordinated by bpe in the crystallographic literature (Groom *et al.*, 2016 ▸), namely [Cd(S_2_CNEt_2_)_2_(μ-bpe)]_*n*_, which is a linear coordination polymer with a *trans*-N_2_S_4_ donor set (Chai *et al.*, 2003 ▸). Reflecting the smaller size of zinc compared to cadmium, the zinc analogues are binuclear zero-dimensional with bpe bridging two five-coordinate (NS_4_) zinc atoms (Arman *et al.*, 2009 ▸). Even in the presence of excess bpe, the [Zn(S_2_CNEt_2_)_2_]_2_(μ-bpe) species still forms with non-coordinating bpe included in the structure (Lai & Tiekink, 2003 ▸). For the analogous xanthate structures, luminescent, zero-dimensional [Zn(S_2_COCyEt)_2_]_2_(μ-bpe) and one-dimensional [Zn(S_2_COEt)_2_(μ-bpe)]_*n*_ are formed with the dimensionality correlated with the steric bulk of the xanthate-bound *R* groups (Kang *et al.*, 2010 ▸). With the sterically unencumbered cadmium di­thio­phosphate analogues, linear coordination polymers are formed regardless of the size of *R*, *i.e*. for {Cd[S_2_P(O*R*)_2_]_2_(μ-bpe)}_*n*_, *R* = *i*Pr and Cy (Lai & Tiekink, 2004 ▸).

There are literature precedents for both bidentate, bridging and monodentate bpe ligands in cadmium structures as observed in (I), *i.e*. [Cd(NO_3_)(μ_2_-NO_3_)(μ-bpe)(bpe)(OH_2_)]_*n*_ (Dong *et al.*, 1999 ▸) and [Cd_2_(SSO_3_)_2_(μ-bpe)(bpe)_2_(OH_2_)_4_]_*n*_ (Paul *et al.*, 2011 ▸). Another structure has both bridging and monodentate bpe ligands as well as non-coordinating bpe ligands (and non-coordinating 4,4′-bipyrid­yl), *i.e*. [Cd(NO_3_)(μ-bpe)(bpe)_2_(OH_2_)_2_]NO_3_(bpe)(4,4′-bipyrid­yl)(H_2_O)_4.45_ (Lu *et al.*, 2001 ▸).

## Synthesis and crystallization   

The title compound was isolated regardless of the ratio, *i.e*. 2:1, 1:1 or 1:2, between the precursor mol­ecules. In a typical experiment, Cd[S_2_CN(iPr)CH_2_CH_2_OH]_2_ (190 mg, 0.50 mmol) was dissolved in boiling aceto­nitrile (30 ml). *trans*-1,2-Dipyridin-4-yl­ethyl­ene (47 mg, 0.25 mmol) was added to this solution, which was allowed to slowly cool to room temperature. Yellow prisms precipitated within an hour. The yield was not measured but was close to qu­anti­tative based on Cd. M.p. = 463–465 K (uncorrected). IR (neat solid, cm^−1^): 1607 *m*, 1449 *ms*, 1407 *s*, 1170 *s*, 1037 *s*, 968 *s*, 954 *s*, 824 *s*. NMR: ^1^H δ (p.p.m.): 8.6 (*dd*, Ar, 1.46 Hz, 4.68 Hz), 7.6 (*dd*, Ar, 1.46 Hz, 4.39 Hz), 7.54 (*s*, –CH=CH–), 5.21 (*sept*., –CH, 6.72 Hz), 4.82 (*t*, –OH, 5.56 Hz), 3.76-3.67 (*m*, –CH_2_–CH_2_–), 1.18 (*d*, CH_3_, 6.72 Hz). TGA: one sharp step (onset at 497 K, mid-point at 502 K, end-point at 509 K; mass loss 62%) followed by a protracted mass loss totalling 71.5%, assigned to decomposition to CdS (calculated mass loss 69.5%).

## Refinement   

Crystal data, data collection and structure refinement details are summarized in Table 6 ▸. The carbon-bound H atoms were placed in calculated positions (C—H = 0.95–1.00 Å) and were included in the refinement in the riding-model approximation, with *U*_iso_(H) set to 1.2–1.5*U*_eq_(C). The oxygen-bound H atoms were located in a difference Fourier map but were refined with a distance restraint of O—H = 0.84±0.01 Å, and with *U*_iso_(H) set to 1.5*U*_eq_(O).

## Supplementary Material

Crystal structure: contains datablock(s) I, global. DOI: 10.1107/S2056989016010768/hb7594sup1.cif

Structure factors: contains datablock(s) I. DOI: 10.1107/S2056989016010768/hb7594Isup2.hkl

CCDC reference: 1489732

Additional supporting information: crystallographic information; 3D view; checkCIF report

## Figures and Tables

**Table 1:** Selected geometric parameters (Å, °)

Cd—S1	2.6019 (8)	Cd—N4	2.454 (3)
Cd—S2	2.7457 (8)	C1—S1	1.726 (3)
Cd—S3	2.6043 (8)	C1—S2	1.717 (3)
Cd—S4	2.6967 (8)	C7—S3	1.721 (3)
Cd—N3	2.439 (3)	C7—S4	1.727 (3)
			
S1—Cd—S2	67.31 (2)	S2—Cd—N4	152.04 (6)
S1—Cd—S3	178.06 (3)	S3—Cd—S4	68.00 (2)
S1—Cd—S4	112.08 (3)	S3—Cd—N3	86.63 (7)
S1—Cd—N3	93.39 (7)	S3—Cd—N4	95.94 (6)
S1—Cd—N4	85.98 (6)	S4—Cd—N3	154.39 (6)
S2—Cd—S3	110.75 (3)	S4—Cd—N4	97.72 (6)
S2—Cd—S4	99.85 (3)	N3—Cd—N4	80.87 (9)
S2—Cd—N3	92.19 (7)		

**Table 2:** Hydrogen-bond geometry (Å, °)

*D*—H⋯*A*	*D*—H	H⋯*A*	*D*⋯*A*	*D*—H⋯*A*
O1—H1*O*⋯N5^i^	0.83 (4)	1.82 (4)	2.655 (4)	177 (3)
O2—H2*O*⋯O1^ii^	0.84 (3)	1.87 (3)	2.689 (3)	165 (5)
C25—H25⋯O2^iii^	0.95	2.44	3.261 (4)	145
C28—H28⋯N7^iv^	0.95	2.56	3.296 (7)	134

Symmetry codes: (i) 
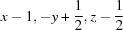
; (ii) 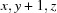; (iii) 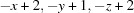; (iv) 
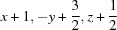
.

**Table 3:** Summary of short inter­atomic contacts (Å)

Contact	Distance	Symmetry
S1⋯H6*A*	2.98	2 − *x*, −*y*, 2 − *z*
S4⋯H18	2.97	2 − *x*, 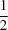 + *y*, 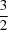 − *z*
O1⋯H11*C*	2.64	*x*, −1 + *y*, *z*
O2⋯H30	2.66	2 − *x*, 1 − *y*, 2 − *z*
C1⋯H20	2.83	2 − *x*, − 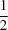 + *y*, 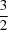 − *z*
C3⋯H2*O*	2.69 (3)	*x*, −1 + *y*, *z*
C24⋯H3*A*	2.72	2 − *x*, − 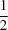 + *y*, 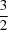 − *z*
C28⋯H1*O*	2.83 (3)	1 + *x*, 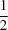 − *y*, 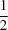 + *z*
C29⋯H1*O*	2.67 (4)	1 + *x*, 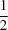 − *y*, 1 + *z*
C16⋯C19	3.245 (4)	2 − *x*, − 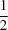 + *y*, 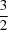 − *z*
C16⋯C20	3.377 (5)	2 − *x*, − 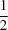 + *y*, 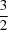 − *z*
H5*A*⋯H23	2.31	2 − *x*, −*y*, 2 − *z*

**Table 4:** Percentage contribution of the different inter­molecular contacts to the Hirshfeld surface

Contact	Contribution
H⋯H	51.9
O⋯H/H⋯O	6.0
C⋯H/H⋯C	15.9
N⋯H/H⋯N	10.6
C⋯C	3.1
S⋯H/H⋯S	10.3
C⋯S/S⋯C	0.8
N⋯S/S⋯N	0.7
C⋯N/N⋯C	0.7

**Table 5:** Enrichment ratios (ER)

Contact	ER
H⋯H	0.97
O⋯H/H⋯O	1.36
N⋯H/H⋯N	1.20
C⋯C	2.23
S⋯H/H⋯S	1.19
C⋯H/H⋯C	0.92
C⋯S/S⋯C	0.58
C⋯N/N⋯C	0.49

**Table 6:** Experimental details

Crystal data	
Chemical formula	[Cd_2_(C_12_H_10_N_2_)_3_(C_6_H_12_NOS_2_)_4_]·4C_2_H_3_N
*M*_r_	1648.84
Crystal system, space group	Monoclinic, *P*2_1_/*c*
Temperature (K)	153
*a*, *b*, *c* (Å)	16.884 (2), 14.4021 (15), 17.327 (2)
β (°)	109.112 (3)
*V* (Å^3^)	3981.0 (8)
*Z*	2
Radiation type	Mo *K*α
μ (mm^−1^)	0.80
Crystal size (mm)	0.35 × 0.25 × 0.10

Data collection	
Diffractometer	AFC12K/SATURN724
Absorption correction	Multi-scan (*ABSCOR*; Higashi, 1995 ▸)
*T*_min_, *T*_max_	0.752, 1.000
No. of measured, independent and observed [*I* > 2σ(*I*)] reflections	36088, 8240, 7736
*R*_int_	0.033
(sin θ/λ)_max_ (Å^−1^)	0.628

Refinement	
*R*[*F* ^2^ > 2σ(*F* ^2^)], *wR*(*F* ^2^), *S*	0.047, 0.115, 1.13
No. of reflections	8240
No. of parameters	445
No. of restraints	2
Δρ_max_, Δρ_min_ (e Å^−3^)	1.43, −0.81

Computer programs: *CrystalClear* (Molecular Structure Corporation & Rigaku, 2005 ▸), *SHELXS97* (Sheldrick, 2008 ▸), *SHELXL2014* (Sheldrick, 2015 ▸), *ORTEP-3 for Windows* (Farrugia, 2012 ▸), *DIAMOND* (Brandenburg, 2006 ▸) and *publCIF* (Westrip, 2010 ▸).

## supplementary crystallographic information

### Crystal data

**Table:**

[Cd_2_(C_12_H_10_N_2_)_3_(C_6_H_12_NOS_2_)_4_]·4C_2_H_3_N	*F*(000) = 1704
*M**_r_* = 1648.84	*D*_x_ = 1.376 Mg m^−^^3^
Monoclinic, *P*2_1_/*c*	Mo *K*α radiation, λ = 0.71073 Å
*a* = 16.884 (2) Å	Cell parameters from 15613 reflections
*b* = 14.4021 (15) Å	θ = 2.5–30.5°
*c* = 17.327 (2) Å	µ = 0.80 mm^−^^1^
β = 109.112 (3)°	*T* = 153 K
*V* = 3981.0 (8) Å^3^	Prism, yellow
*Z* = 2	0.35 × 0.25 × 0.10 mm

### Data collection

**Table:**

AFC12K/SATURN724 diffractometer	8240 independent reflections
Radiation source: fine-focus sealed tube	7736 reflections with *I* > 2σ(*I*)
Graphite monochromator	*R*_int_ = 0.033
ω scans	θ_max_ = 26.5°, θ_min_ = 2.0°
Absorption correction: multi-scan (ABSCOR; Higashi, 1995)	*h* = −21→19
*T*_min_ = 0.752, *T*_max_ = 1.000	*k* = −18→18
36088 measured reflections	*l* = −21→21

### Refinement

**Table:**

Refinement on *F*^2^	2 restraints
Least-squares matrix: full	Hydrogen site location: mixed
*R*[*F*^2^ > 2σ(*F*^2^)] = 0.047	*w* = 1/[σ^2^(*F*_o_^2^) + (0.0538*P*)^2^ + 4.1818*P*] where *P* = (*F*_o_^2^ + 2*F*_c_^2^)/3
*wR*(*F*^2^) = 0.115	(Δ/σ)_max_ = 0.002
*S* = 1.13	Δρ_max_ = 1.43 e Å^−^^3^
8240 reflections	Δρ_min_ = −0.81 e Å^−^^3^
445 parameters	

### Special details

**Table:**

Geometry. All esds (except the esd in the dihedral angle between two l.s. planes) are estimated using the full covariance matrix. The cell esds are taken into account individually in the estimation of esds in distances, angles and torsion angles; correlations between esds in cell parameters are only used when they are defined by crystal symmetry. An approximate (isotropic) treatment of cell esds is used for estimating esds involving l.s. planes.

### Fractional atomic coordinates and isotropic or equivalent isotropic displacement parameters (Å^2^)

**Table:**

	*x*	*y*	*z*	*U*_iso_*/*U*_eq_	
Cd	0.86121 (2)	0.27028 (2)	0.77182 (2)	0.02811 (9)	
S1	0.91301 (5)	0.14219 (6)	0.88291 (5)	0.0409 (2)	
S2	0.74876 (5)	0.12713 (5)	0.75128 (4)	0.03067 (16)	
S3	0.80475 (5)	0.39522 (5)	0.65839 (5)	0.03553 (18)	
S4	0.77645 (5)	0.40603 (5)	0.81826 (4)	0.03206 (17)	
O1	0.65163 (14)	−0.17072 (16)	0.73753 (15)	0.0409 (6)	
H1O	0.6124 (19)	−0.142 (3)	0.704 (2)	0.061*	
O2	0.70145 (16)	0.70048 (15)	0.85595 (14)	0.0390 (5)	
H2O	0.677 (3)	0.738 (2)	0.8189 (19)	0.059*	
N1	0.81603 (16)	−0.00780 (17)	0.85909 (15)	0.0320 (5)	
N2	0.71824 (16)	0.53113 (17)	0.69907 (15)	0.0315 (5)	
N3	0.92734 (16)	0.19783 (18)	0.68118 (16)	0.0338 (6)	
N4	1.00214 (16)	0.33683 (17)	0.82938 (15)	0.0309 (5)	
N5	1.53044 (18)	0.5788 (2)	1.12686 (18)	0.0427 (7)	
N6	0.4531 (4)	0.6223 (6)	0.6589 (4)	0.144 (3)	
N7	0.4216 (5)	0.7244 (4)	0.4145 (4)	0.121 (2)	
C1	0.82450 (19)	0.07842 (19)	0.83301 (18)	0.0295 (6)	
C2	0.7352 (2)	−0.0575 (2)	0.82882 (19)	0.0352 (7)	
H2A	0.6892	−0.0117	0.8191	0.042*	
H2B	0.7311	−0.1006	0.8719	0.042*	
C3	0.7226 (2)	−0.1119 (2)	0.7513 (2)	0.0373 (7)	
H3A	0.7136	−0.0688	0.7047	0.045*	
H3B	0.7730	−0.1497	0.7564	0.045*	
C4	0.8842 (2)	−0.0533 (2)	0.92667 (19)	0.0345 (7)	
H4	0.9366	−0.0166	0.9349	0.041*	
C5	0.9014 (2)	−0.1517 (2)	0.9049 (2)	0.0408 (7)	
H5A	0.9520	−0.1753	0.9465	0.061*	
H5B	0.9097	−0.1518	0.8515	0.061*	
H5C	0.8537	−0.1915	0.9028	0.061*	
C6	0.8641 (3)	−0.0487 (3)	1.0054 (2)	0.0517 (9)	
H6A	0.9121	−0.0716	1.0504	0.078*	
H6B	0.8149	−0.0872	1.0006	0.078*	
H6C	0.8524	0.0158	1.0162	0.078*	
C7	0.76189 (18)	0.45215 (19)	0.72260 (18)	0.0283 (6)	
C8	0.6803 (2)	0.5800 (2)	0.75284 (19)	0.0338 (7)	
H8A	0.6565	0.5338	0.7814	0.041*	
H8B	0.6337	0.6194	0.7191	0.041*	
C9	0.7430 (2)	0.6407 (2)	0.8161 (2)	0.0361 (7)	
H9A	0.7831	0.6006	0.8571	0.043*	
H9B	0.7752	0.6784	0.7889	0.043*	
C10	0.6999 (2)	0.5689 (2)	0.6145 (2)	0.0426 (8)	
H10	0.7369	0.5350	0.5892	0.051*	
C11	0.7209 (3)	0.6707 (3)	0.6145 (3)	0.0628 (11)	
H11A	0.7093	0.6920	0.5581	0.094*	
H11B	0.7803	0.6801	0.6453	0.094*	
H11C	0.6866	0.7062	0.6401	0.094*	
C12	0.6102 (3)	0.5471 (4)	0.5636 (2)	0.0681 (12)	
H12A	0.5997	0.5692	0.5076	0.102*	
H12B	0.5719	0.5782	0.5872	0.102*	
H12C	0.6012	0.4799	0.5630	0.102*	
C13	0.8862 (2)	0.1867 (2)	0.6011 (2)	0.0416 (8)	
H13	0.8344	0.2182	0.5779	0.050*	
C14	0.9149 (2)	0.1318 (2)	0.5505 (2)	0.0409 (8)	
H14	0.8836	0.1268	0.4941	0.049*	
C15	0.9902 (2)	0.0840 (2)	0.58273 (19)	0.0338 (7)	
C16	1.0352 (2)	0.0992 (2)	0.6648 (2)	0.0351 (7)	
H16	1.0886	0.0712	0.6888	0.042*	
C17	1.0018 (2)	0.1548 (2)	0.7110 (2)	0.0359 (7)	
H17	1.0331	0.1633	0.7671	0.043*	
C18	1.0222 (2)	0.0174 (2)	0.53632 (19)	0.0355 (7)	
H18	1.0787	−0.0024	0.5596	0.043*	
C19	1.0297 (2)	0.4016 (2)	0.78886 (19)	0.0331 (6)	
H19	0.9938	0.4200	0.7364	0.040*	
C20	1.10796 (19)	0.4431 (2)	0.81939 (19)	0.0338 (6)	
H20	1.1244	0.4887	0.7881	0.041*	
C21	1.16227 (19)	0.4178 (2)	0.89581 (18)	0.0304 (6)	
C22	1.13422 (19)	0.3486 (2)	0.93706 (18)	0.0334 (6)	
H22	1.1696	0.3271	0.9887	0.040*	
C23	1.0553 (2)	0.3117 (2)	0.90285 (18)	0.0337 (6)	
H23	1.0374	0.2658	0.9329	0.040*	
C24	1.2428 (2)	0.4655 (2)	0.93038 (19)	0.0337 (6)	
H24	1.2517	0.5206	0.9042	0.040*	
C25	1.3045 (2)	0.4366 (2)	0.99626 (19)	0.0356 (7)	
H25	1.2965	0.3788	1.0190	0.043*	
C26	1.38345 (19)	0.4854 (2)	1.03704 (19)	0.0339 (6)	
C27	1.4043 (2)	0.5708 (2)	1.0115 (2)	0.0392 (7)	
H27	1.3686	0.5987	0.9629	0.047*	
C28	1.4775 (2)	0.6149 (3)	1.0576 (2)	0.0440 (8)	
H28	1.4908	0.6732	1.0394	0.053*	
C29	1.5114 (2)	0.4955 (3)	1.1492 (2)	0.0430 (8)	
H29	1.5492	0.4681	1.1970	0.052*	
C30	1.4403 (2)	0.4469 (2)	1.1072 (2)	0.0383 (7)	
H30	1.4299	0.3875	1.1258	0.046*	
C31	0.3930 (3)	0.6527 (4)	0.6166 (3)	0.0811 (16)	
C32	0.3169 (3)	0.6944 (4)	0.5636 (3)	0.0715 (13)	
H32A	0.3086	0.7549	0.5857	0.107*	
H32B	0.2692	0.6539	0.5603	0.107*	
H32C	0.3210	0.7027	0.5090	0.107*	
C33	0.4380 (4)	0.6986 (4)	0.3603 (3)	0.0757 (14)	
C34	0.4564 (4)	0.6632 (6)	0.2899 (4)	0.119 (3)	
H34A	0.4786	0.5999	0.3012	0.178*	
H34B	0.4980	0.7031	0.2782	0.178*	
H34C	0.4049	0.6624	0.2426	0.178*	

### Atomic displacement parameters (Å^2^)

**Table:**

	*U*^11^	*U*^22^	*U*^33^	*U*^12^	*U*^13^	*U*^23^
Cd	0.02812 (14)	0.02260 (13)	0.03125 (13)	0.00156 (7)	0.00653 (10)	0.00010 (8)
S1	0.0366 (4)	0.0298 (4)	0.0430 (4)	−0.0089 (3)	−0.0052 (4)	0.0069 (3)
S2	0.0283 (4)	0.0252 (3)	0.0334 (4)	0.0014 (3)	0.0032 (3)	0.0011 (3)
S3	0.0419 (5)	0.0318 (4)	0.0335 (4)	0.0103 (3)	0.0132 (3)	0.0037 (3)
S4	0.0373 (4)	0.0265 (3)	0.0312 (4)	0.0030 (3)	0.0096 (3)	0.0016 (3)
O1	0.0307 (12)	0.0283 (11)	0.0510 (14)	−0.0040 (9)	−0.0041 (10)	0.0085 (10)
O2	0.0532 (15)	0.0263 (11)	0.0402 (12)	0.0070 (10)	0.0189 (11)	0.0020 (9)
N1	0.0309 (14)	0.0261 (12)	0.0331 (12)	−0.0038 (10)	0.0025 (11)	0.0019 (10)
N2	0.0324 (14)	0.0270 (12)	0.0335 (12)	0.0059 (10)	0.0086 (11)	0.0034 (10)
N3	0.0322 (14)	0.0327 (13)	0.0352 (13)	0.0048 (11)	0.0094 (11)	−0.0044 (11)
N4	0.0306 (13)	0.0261 (12)	0.0353 (13)	−0.0009 (10)	0.0096 (11)	−0.0012 (10)
N5	0.0317 (15)	0.0477 (16)	0.0436 (15)	−0.0062 (12)	0.0053 (12)	−0.0076 (13)
N6	0.081 (4)	0.252 (8)	0.111 (4)	0.064 (5)	0.049 (3)	0.090 (5)
N7	0.174 (7)	0.100 (4)	0.112 (4)	−0.036 (4)	0.076 (5)	−0.044 (3)
C1	0.0322 (16)	0.0232 (13)	0.0315 (14)	−0.0017 (11)	0.0084 (12)	−0.0016 (11)
C2	0.0311 (16)	0.0319 (15)	0.0394 (16)	−0.0069 (12)	0.0071 (13)	0.0028 (13)
C3	0.0311 (17)	0.0302 (15)	0.0445 (17)	−0.0047 (13)	0.0041 (14)	0.0037 (13)
C4	0.0309 (16)	0.0303 (15)	0.0366 (16)	0.0001 (12)	0.0034 (13)	0.0032 (13)
C5	0.046 (2)	0.0329 (16)	0.0393 (16)	0.0094 (14)	0.0086 (15)	0.0055 (14)
C6	0.055 (2)	0.060 (2)	0.0389 (17)	0.0110 (19)	0.0140 (17)	0.0000 (17)
C7	0.0239 (14)	0.0227 (13)	0.0340 (14)	−0.0010 (11)	0.0035 (12)	−0.0015 (11)
C8	0.0347 (17)	0.0265 (14)	0.0404 (16)	0.0058 (12)	0.0125 (14)	0.0007 (13)
C9	0.0349 (17)	0.0283 (15)	0.0435 (17)	0.0034 (13)	0.0110 (14)	−0.0024 (13)
C10	0.052 (2)	0.0368 (17)	0.0389 (17)	0.0166 (15)	0.0155 (16)	0.0121 (14)
C11	0.091 (3)	0.044 (2)	0.061 (2)	0.011 (2)	0.035 (2)	0.0156 (19)
C12	0.065 (3)	0.083 (3)	0.043 (2)	0.010 (2)	−0.001 (2)	0.007 (2)
C13	0.044 (2)	0.0375 (17)	0.0379 (16)	0.0130 (15)	0.0069 (15)	−0.0028 (14)
C14	0.048 (2)	0.0373 (17)	0.0332 (16)	0.0086 (15)	0.0073 (15)	−0.0029 (13)
C15	0.0381 (17)	0.0267 (14)	0.0390 (16)	0.0030 (12)	0.0157 (14)	0.0035 (13)
C16	0.0289 (16)	0.0320 (15)	0.0438 (17)	0.0026 (12)	0.0111 (14)	−0.0023 (13)
C17	0.0317 (16)	0.0345 (16)	0.0381 (16)	0.0011 (13)	0.0068 (13)	−0.0055 (13)
C18	0.0372 (17)	0.0322 (15)	0.0394 (15)	0.0036 (13)	0.0156 (14)	0.0050 (13)
C19	0.0329 (16)	0.0317 (15)	0.0321 (14)	−0.0006 (12)	0.0072 (13)	0.0017 (12)
C20	0.0324 (16)	0.0313 (15)	0.0367 (15)	−0.0019 (12)	0.0102 (13)	0.0044 (13)
C21	0.0267 (15)	0.0291 (14)	0.0338 (14)	−0.0027 (11)	0.0077 (12)	−0.0039 (12)
C22	0.0300 (16)	0.0342 (15)	0.0311 (14)	−0.0032 (12)	0.0032 (12)	−0.0005 (12)
C23	0.0340 (17)	0.0317 (15)	0.0321 (15)	−0.0030 (12)	0.0066 (13)	0.0032 (12)
C24	0.0340 (17)	0.0304 (15)	0.0369 (15)	−0.0042 (12)	0.0119 (13)	0.0000 (13)
C25	0.0335 (17)	0.0361 (16)	0.0356 (15)	−0.0045 (13)	0.0093 (13)	−0.0001 (13)
C26	0.0298 (16)	0.0357 (15)	0.0360 (15)	−0.0051 (12)	0.0106 (13)	−0.0049 (13)
C27	0.0317 (17)	0.0404 (17)	0.0398 (17)	−0.0012 (14)	0.0039 (14)	0.0015 (14)
C28	0.0363 (19)	0.0414 (18)	0.0506 (19)	−0.0076 (14)	0.0090 (16)	−0.0005 (16)
C29	0.0334 (18)	0.056 (2)	0.0350 (16)	−0.0043 (15)	0.0050 (14)	0.0002 (15)
C30	0.0328 (17)	0.0418 (18)	0.0392 (16)	−0.0061 (14)	0.0106 (14)	0.0020 (14)
C31	0.065 (3)	0.119 (5)	0.070 (3)	0.016 (3)	0.037 (3)	0.033 (3)
C32	0.057 (3)	0.093 (4)	0.060 (3)	−0.002 (3)	0.014 (2)	0.014 (3)
C33	0.084 (4)	0.068 (3)	0.076 (3)	−0.018 (3)	0.028 (3)	−0.015 (3)
C34	0.087 (4)	0.193 (8)	0.085 (4)	−0.049 (5)	0.040 (3)	−0.059 (5)

### Geometric parameters (Å, º)

**Table:**

Cd—S1	2.6019 (8)	C10—C12	1.514 (6)
Cd—S2	2.7457 (8)	C10—H10	1.0000
Cd—S3	2.6043 (8)	C11—H11A	0.9800
Cd—S4	2.6967 (8)	C11—H11B	0.9800
Cd—N3	2.439 (3)	C11—H11C	0.9800
Cd—N4	2.454 (3)	C12—H12A	0.9800
C1—S1	1.726 (3)	C12—H12B	0.9800
C1—S2	1.717 (3)	C12—H12C	0.9800
C7—S3	1.721 (3)	C13—C14	1.381 (5)
C7—S4	1.727 (3)	C13—H13	0.9500
O1—C3	1.422 (4)	C14—C15	1.391 (5)
O1—H1O	0.839 (10)	C14—H14	0.9500
O2—C9	1.425 (4)	C15—C16	1.393 (4)
O2—H2O	0.840 (10)	C15—C18	1.464 (4)
N1—C1	1.345 (4)	C16—C17	1.377 (4)
N1—C2	1.477 (4)	C16—H16	0.9500
N1—C4	1.498 (4)	C17—H17	0.9500
N2—C7	1.344 (4)	C18—C18^i^	1.334 (6)
N2—C8	1.472 (4)	C18—H18	0.9500
N2—C10	1.497 (4)	C19—C20	1.388 (4)
N3—C17	1.343 (4)	C19—H19	0.9500
N3—C13	1.343 (4)	C20—C21	1.390 (4)
N4—C19	1.339 (4)	C20—H20	0.9500
N4—C23	1.345 (4)	C21—C22	1.396 (4)
N5—C29	1.333 (5)	C21—C24	1.465 (4)
N5—C28	1.344 (5)	C22—C23	1.375 (4)
N6—C31	1.128 (7)	C22—H22	0.9500
N7—C33	1.126 (7)	C23—H23	0.9500
C2—C3	1.508 (5)	C24—C25	1.335 (4)
C2—H2A	0.9900	C24—H24	0.9500
C2—H2B	0.9900	C25—C26	1.468 (4)
C3—H3A	0.9900	C25—H25	0.9500
C3—H3B	0.9900	C26—C27	1.391 (5)
C4—C6	1.510 (5)	C26—C30	1.393 (5)
C4—C5	1.519 (4)	C27—C28	1.386 (5)
C4—H4	1.0000	C27—H27	0.9500
C5—H5A	0.9800	C28—H28	0.9500
C5—H5B	0.9800	C29—C30	1.375 (5)
C5—H5C	0.9800	C29—H29	0.9500
C6—H6A	0.9800	C30—H30	0.9500
C6—H6B	0.9800	C31—C32	1.443 (7)
C6—H6C	0.9800	C32—H32A	0.9800
C8—C9	1.525 (4)	C32—H32B	0.9800
C8—H8A	0.9900	C32—H32C	0.9800
C8—H8B	0.9900	C33—C34	1.445 (8)
C9—H9A	0.9900	C34—H34A	0.9800
C9—H9B	0.9900	C34—H34B	0.9800
C10—C11	1.510 (5)	C34—H34C	0.9800
			
S1—Cd—S2	67.31 (2)	C11—C10—C12	113.0 (3)
S1—Cd—S3	178.06 (3)	N2—C10—H10	107.0
S1—Cd—S4	112.08 (3)	C11—C10—H10	107.0
S1—Cd—N3	93.39 (7)	C12—C10—H10	107.0
S1—Cd—N4	85.98 (6)	C10—C11—H11A	109.5
S2—Cd—S3	110.75 (3)	C10—C11—H11B	109.5
S2—Cd—S4	99.85 (3)	H11A—C11—H11B	109.5
S2—Cd—N3	92.19 (7)	C10—C11—H11C	109.5
S2—Cd—N4	152.04 (6)	H11A—C11—H11C	109.5
S3—Cd—S4	68.00 (2)	H11B—C11—H11C	109.5
S3—Cd—N3	86.63 (7)	C10—C12—H12A	109.5
S3—Cd—N4	95.94 (6)	C10—C12—H12B	109.5
S4—Cd—N3	154.39 (6)	H12A—C12—H12B	109.5
S4—Cd—N4	97.72 (6)	C10—C12—H12C	109.5
N3—Cd—N4	80.87 (9)	H12A—C12—H12C	109.5
C1—S1—Cd	88.88 (10)	H12B—C12—H12C	109.5
C1—S2—Cd	84.44 (10)	N3—C13—C14	123.6 (3)
C7—S3—Cd	88.21 (10)	N3—C13—H13	118.2
C7—S4—Cd	85.13 (10)	C14—C13—H13	118.2
C3—O1—H1O	104 (3)	C13—C14—C15	119.5 (3)
C9—O2—H2O	102 (3)	C13—C14—H14	120.2
C1—N1—C2	121.0 (3)	C15—C14—H14	120.2
C1—N1—C4	121.8 (2)	C14—C15—C16	117.0 (3)
C2—N1—C4	116.8 (2)	C14—C15—C18	123.8 (3)
C7—N2—C8	121.5 (3)	C16—C15—C18	119.2 (3)
C7—N2—C10	121.4 (3)	C17—C16—C15	119.6 (3)
C8—N2—C10	116.9 (2)	C17—C16—H16	120.2
C17—N3—C13	116.4 (3)	C15—C16—H16	120.2
C17—N3—Cd	121.2 (2)	N3—C17—C16	123.7 (3)
C13—N3—Cd	121.7 (2)	N3—C17—H17	118.2
C19—N4—C23	116.4 (3)	C16—C17—H17	118.2
C19—N4—Cd	121.1 (2)	C18^i^—C18—C15	124.8 (4)
C23—N4—Cd	122.5 (2)	C18^i^—C18—H18	117.6
C29—N5—C28	117.0 (3)	C15—C18—H18	117.6
N1—C1—S2	121.5 (2)	N4—C19—C20	123.4 (3)
N1—C1—S1	119.5 (2)	N4—C19—H19	118.3
S2—C1—S1	119.00 (17)	C20—C19—H19	118.3
N1—C2—C3	114.3 (3)	C19—C20—C21	119.9 (3)
N1—C2—H2A	108.7	C19—C20—H20	120.0
C3—C2—H2A	108.7	C21—C20—H20	120.0
N1—C2—H2B	108.7	C20—C21—C22	116.5 (3)
C3—C2—H2B	108.7	C20—C21—C24	120.1 (3)
H2A—C2—H2B	107.6	C22—C21—C24	123.4 (3)
O1—C3—C2	109.0 (3)	C23—C22—C21	119.9 (3)
O1—C3—H3A	109.9	C23—C22—H22	120.1
C2—C3—H3A	109.9	C21—C22—H22	120.1
O1—C3—H3B	109.9	N4—C23—C22	123.8 (3)
C2—C3—H3B	109.9	N4—C23—H23	118.1
H3A—C3—H3B	108.3	C22—C23—H23	118.1
N1—C4—C6	110.1 (3)	C25—C24—C21	124.3 (3)
N1—C4—C5	112.0 (3)	C25—C24—H24	117.8
C6—C4—C5	112.5 (3)	C21—C24—H24	117.8
N1—C4—H4	107.3	C24—C25—C26	126.5 (3)
C6—C4—H4	107.3	C24—C25—H25	116.8
C5—C4—H4	107.3	C26—C25—H25	116.8
C4—C5—H5A	109.5	C27—C26—C30	117.2 (3)
C4—C5—H5B	109.5	C27—C26—C25	123.7 (3)
H5A—C5—H5B	109.5	C30—C26—C25	119.1 (3)
C4—C5—H5C	109.5	C28—C27—C26	119.5 (3)
H5A—C5—H5C	109.5	C28—C27—H27	120.3
H5B—C5—H5C	109.5	C26—C27—H27	120.3
C4—C6—H6A	109.5	N5—C28—C27	123.0 (3)
C4—C6—H6B	109.5	N5—C28—H28	118.5
H6A—C6—H6B	109.5	C27—C28—H28	118.5
C4—C6—H6C	109.5	N5—C29—C30	123.8 (3)
H6A—C6—H6C	109.5	N5—C29—H29	118.1
H6B—C6—H6C	109.5	C30—C29—H29	118.1
N2—C7—S3	120.8 (2)	C29—C30—C26	119.5 (3)
N2—C7—S4	120.5 (2)	C29—C30—H30	120.3
S3—C7—S4	118.65 (16)	C26—C30—H30	120.3
N2—C8—C9	112.6 (3)	N6—C31—C32	178.2 (9)
N2—C8—H8A	109.1	C31—C32—H32A	109.5
C9—C8—H8A	109.1	C31—C32—H32B	109.5
N2—C8—H8B	109.1	H32A—C32—H32B	109.5
C9—C8—H8B	109.1	C31—C32—H32C	109.5
H8A—C8—H8B	107.8	H32A—C32—H32C	109.5
O2—C9—C8	111.0 (3)	H32B—C32—H32C	109.5
O2—C9—H9A	109.4	N7—C33—C34	177.8 (7)
C8—C9—H9A	109.4	C33—C34—H34A	109.5
O2—C9—H9B	109.4	C33—C34—H34B	109.5
C8—C9—H9B	109.4	H34A—C34—H34B	109.5
H9A—C9—H9B	108.0	C33—C34—H34C	109.5
N2—C10—C11	112.3 (3)	H34A—C34—H34C	109.5
N2—C10—C12	110.1 (3)	H34B—C34—H34C	109.5
			
C2—N1—C1—S2	−11.5 (4)	C13—C14—C15—C16	−3.7 (5)
C4—N1—C1—S2	175.8 (2)	C13—C14—C15—C18	174.0 (3)
C2—N1—C1—S1	168.1 (2)	C14—C15—C16—C17	4.0 (5)
C4—N1—C1—S1	−4.6 (4)	C18—C15—C16—C17	−173.9 (3)
Cd—S2—C1—N1	−174.7 (3)	C13—N3—C17—C16	−2.1 (5)
Cd—S2—C1—S1	5.71 (16)	Cd—N3—C17—C16	168.2 (2)
Cd—S1—C1—N1	174.4 (2)	C15—C16—C17—N3	−1.1 (5)
Cd—S1—C1—S2	−6.00 (17)	C14—C15—C18—C18^i^	−12.2 (6)
C1—N1—C2—C3	87.7 (4)	C16—C15—C18—C18^i^	165.5 (4)
C4—N1—C2—C3	−99.3 (3)	C23—N4—C19—C20	−0.9 (4)
N1—C2—C3—O1	168.4 (2)	Cd—N4—C19—C20	178.4 (2)
C1—N1—C4—C6	101.6 (3)	N4—C19—C20—C21	0.1 (5)
C2—N1—C4—C6	−71.4 (4)	C19—C20—C21—C22	1.5 (4)
C1—N1—C4—C5	−132.3 (3)	C19—C20—C21—C24	−176.2 (3)
C2—N1—C4—C5	54.6 (4)	C20—C21—C22—C23	−2.2 (4)
C8—N2—C7—S3	179.2 (2)	C24—C21—C22—C23	175.4 (3)
C10—N2—C7—S3	4.5 (4)	C19—N4—C23—C22	0.1 (5)
C8—N2—C7—S4	−1.1 (4)	Cd—N4—C23—C22	−179.2 (2)
C10—N2—C7—S4	−175.8 (2)	C21—C22—C23—N4	1.5 (5)
Cd—S3—C7—N2	−179.5 (2)	C20—C21—C24—C25	−168.5 (3)
Cd—S3—C7—S4	0.81 (16)	C22—C21—C24—C25	13.9 (5)
Cd—S4—C7—N2	179.5 (2)	C21—C24—C25—C26	−174.9 (3)
Cd—S4—C7—S3	−0.78 (15)	C24—C25—C26—C27	0.4 (5)
C7—N2—C8—C9	81.4 (3)	C24—C25—C26—C30	177.9 (3)
C10—N2—C8—C9	−103.6 (3)	C30—C26—C27—C28	−2.4 (5)
N2—C8—C9—O2	168.5 (2)	C25—C26—C27—C28	175.1 (3)
C7—N2—C10—C11	−131.1 (3)	C29—N5—C28—C27	2.3 (5)
C8—N2—C10—C11	53.9 (4)	C26—C27—C28—N5	0.0 (5)
C7—N2—C10—C12	102.0 (4)	C28—N5—C29—C30	−2.2 (5)
C8—N2—C10—C12	−72.9 (4)	N5—C29—C30—C26	−0.2 (5)
C17—N3—C13—C14	2.4 (5)	C27—C26—C30—C29	2.5 (5)
Cd—N3—C13—C14	−167.9 (3)	C25—C26—C30—C29	−175.1 (3)
N3—C13—C14—C15	0.6 (6)		

Symmetry code: (i) −*x*+2, −*y*, −*z*+1.

### Hydrogen-bond geometry (Å, º)

**Table:**

*D*—H···*A*	*D*—H	H···*A*	*D*···*A*	*D*—H···*A*
O1—H1*O*···N5^ii^	0.83 (4)	1.82 (4)	2.655 (4)	177 (3)
O2—H2*O*···O1^iii^	0.84 (3)	1.87 (3)	2.689 (3)	165 (5)
C25—H25···O2^iv^	0.95	2.44	3.261 (4)	145
C28—H28···N7^v^	0.95	2.56	3.296 (7)	134

Symmetry codes: (ii) *x*−1, −*y*+1/2, *z*−1/2; (iii) *x*, *y*+1, *z*; (iv) −*x*+2, −*y*+1, −*z*+2; (v) *x*+1, −*y*+3/2, *z*+1/2.
